# Leber’s Hereditary Optic Neuropathy – Reply Letter to the Editor

**Published:** 2020

**Authors:** Cristina Manole, Bogdana Tăbăcaru, Simona Stanca, Horia Tudor Stanca

**Affiliations:** * “Prof. Dr. Agrippa Ionescu” Clinical Emergency Hospital, Bucharest, Romania; ** “Carol Davila” University of Medicine and Pharmacy Bucharest, Romania

We have read with interest the article by Finsterer J. et al. [**1**], written in response to our LHON case report [**2**]. Rather disappointingly, they have overlooked the purpose and the content of our work and so, we have the following comments.

As our title indicates, our article is a case report, not a clinical trial. The World Health Organization defines the clinical trial as any research study that prospectively assigns human participants or groups of humans to one or more health-related interventions to evaluate the effects on health outcomes [**3**]. A case report is a detailed report of the symptoms, signs, diagnosis, treatment, and follow-up of an individual patient [**4**]. Therefore, our article is not a study of any kind.

**Fig. 1:**
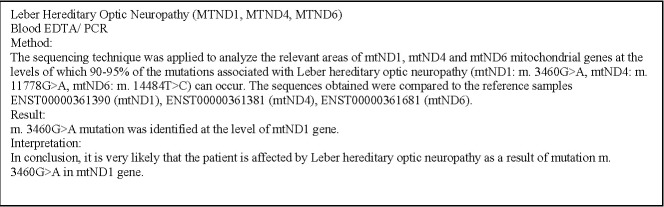
Blood mitochondrial analysis of our patient

By far the most important issue to be addressed is that the diagnosis was genetically confirmed. Blood mitochondrial analysis was performed (**Fig. 1**), being tested the three most common mutations (mtND1: m.3460G>A, mtND4: m.11778G>A, mtND6: m14484T>C), the obtained sequences being compared with the reference sequences (ENST00000361390 mtND1, ENST00000361381 mtND4, ENST00000361681 mtND6). The m.3460G>A mutation in the mtND1 gene was identified in our patient, as it is specified in both abstract and article [**2**]. 

**Fig. 2:**
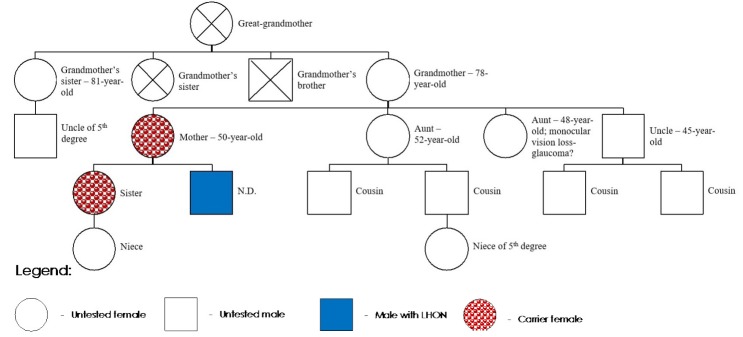
Pedigree

Furthermore, we considered it necessary that the mother was genetically tested, given that “de novo mutation” is rare in LHON, but possible [**5**]. As the mother demonstrated to be positive for m.3460G>A mutation, we also considered the sister to be tested, since she could transmit the mutation to her child. After a rigorous ophthalmological examination, we concluded that both were healthy carriers of the m.3460G>A mutation and were periodically evaluated to detect any early signs of the disease (**Fig. 2**). 

Regarding the mutation load, the proportion of mutant mtDNA and the incidence of disease development do not necessarily show a high association. Variable expression of LHON has been considered to result from a variation in additional genetic or environmental factors other than the level of heteroplasmy. However, a correlation between the extent of heteroplasmy in the circulating leukocytes and the risk of developing optic atrophy or the severity of the disease has also been reported. Thus, the accurate quantification of heteroplasmy will help determine the relative risk of disease expression, although heteroplasmy alone cannot determine the risk of developing optic atrophy in LHON [**6**]. To the best of our knowledge, in Romania, the determination of the level of heteroplasmy in blood leukocytes of LHON patients and unaffected carriers is not routinely performed in clinical practice, in the public health system, or in the private one. No specific environmental precipitant for vision loss in LHON mutation carriers has been clearly identified. Various other systemic illnesses, medications, and toxins have been proposed as triggers for vision loss in the setting of LHON mutations and nutritional deficiencies (e.g. vitamin B12 deficiency) might also play a role in the disease expression through an insufficiency of important metabolic cofactor, but none of these risk factors has been identified in our patients [**7**]. Patients are strongly advised to moderate their alcohol intake and not to smoke, in order to minimize mitochondrial stress [**8**]. The gender could also result from a combination of subtle anatomic, hormonal, and/ or physiologic variations between males and females, the ratio between female and male in symptomatic patients being 1:3 [**5**,**9**]. Due to difficulties in tracing back a more extensive family history, other members of the family could not be genetically tested.

**Fig. 3:**
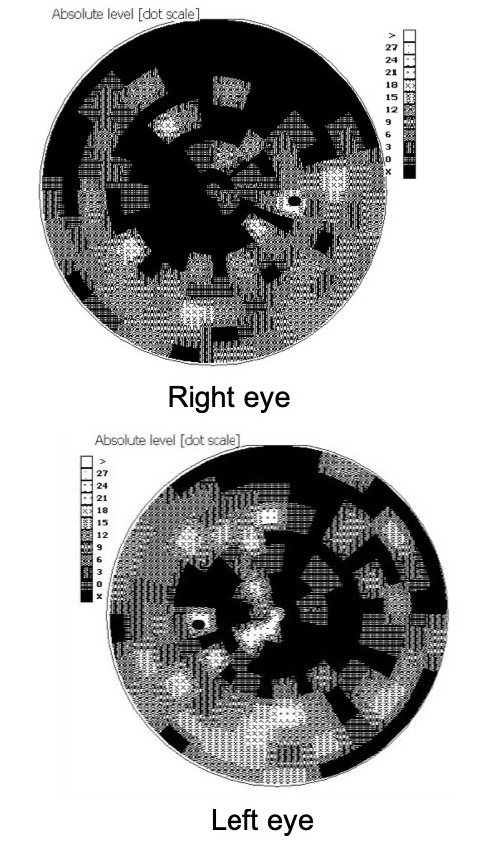
Scotoma in the centrocecal area at initiation of idebenone

**Fig. 4:**
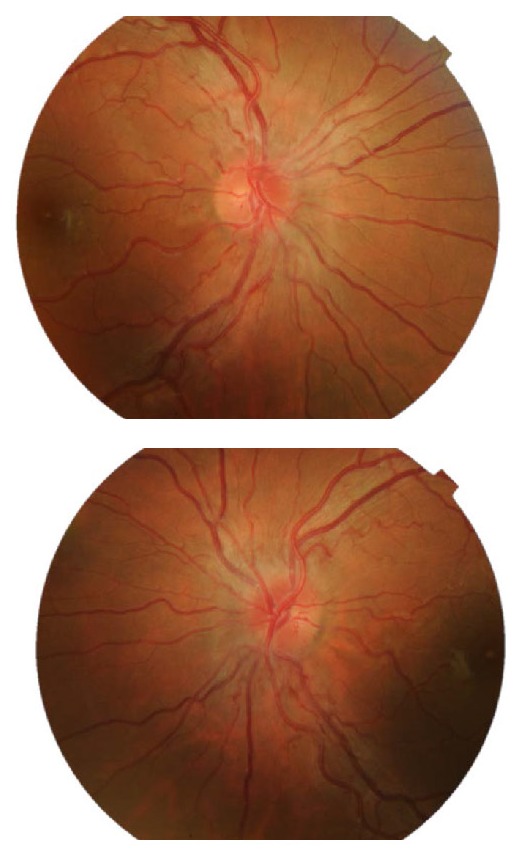
Fundus examination of both eyes at presentation

**Fig. 5:**
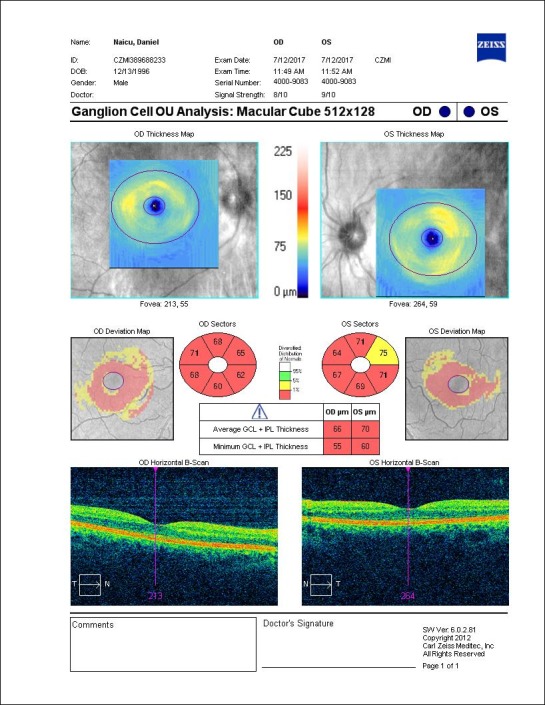
Severely thinned GCL-IPL complex at presentation

Another very important aspect that needs to be specified is that treatment with idebenone was initiated in our patient only after the diagnosis was genetically confirmed [**2**]. Moreover, idebenone treatment in our country is initiated only in patients included in the National Health Program for LHON, our patient satisfying all the criteria for inclusion in the program [**10**]: 

- The non-painful, generally subacute/ acute onset of decreased visual acuity;

- The presence of a central/ centrocecal scotoma, either unilaterally or bilaterally (**Fig. 3**);

- Decreased visual acuity under logMAR 1.0 (ETDRS), within the first 12 months after clinical onset;

- Alteration of color perception (discromatopsy), especially on the red-green axis; 

- Lack of response to glucocorticoid treatment after 15-30 days of treatment; 

- Occurrence of a pseudoedema in the optic disc, affecting retinal ganglion cells (RCGs) and their axons (**Fig. 4**,**5**);

- Positive genetic testing by the appearance of a point mutation in the mitochondrial DNA (**Fig. 1**).

Furthermore, by the time our article [**2**] was published, the patient was under treatment with idebenone for 12 months. However, please note that the patient, who was ophthalmologically evaluated every three months, continued treatment in accordance with the treatment protocol of the National Health Program [**10**] in which he was included. Thus, our goal was not to conduct a trial with idebenone for 12 months, but to report a confirmed m.3460G>A LHON case, non-responder to idebenone treatment. As the frequency of follow-up for affected individuals varies depending on the individual’s personal circumstances and the availability of local health care [**9**], quantification of the oxidative stress or the amount of ATP production for monitoring the therapeutic effect of idebenone is not part of the patient monitoring protocol in our country (visual acuity testing, color perception and visual field) [**10**].

Also, a multidisciplinary approach for our patient was considered and developed between the initial presentation and the genetic confirmation. This approach aimed both outlining a differential diagnosis and a full systemic evaluation in the context of the strong suspicion of LHON. As such, paraclinical investigations were carried out and all extraocular features were excluded [**2**]. 

**Fig. 6:**
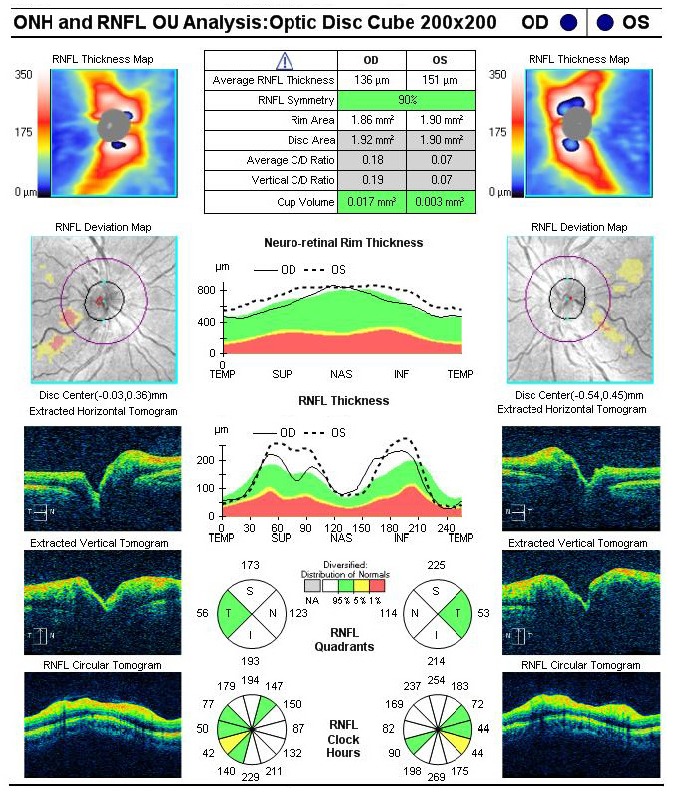
Bilateral increase of the RNFL thickness at presentation

Additionally, the observation of RNFL thickening (**Fig. 6**) in the early stages of the disease was explained in the article [**2**]. It was consistent with both the fundus examination (**Fig. 4**), which demonstrated bilateral protruding, hyperemic, with blurred margins in the nasal quadrant papilla and reduced excavation, and the compensatory increase of mitochondrial biogenesis and/ or axonal stasis along the fibers, which did not allow detection of optic nerve atrophy in the early stages of the disease [**11**].

Finally, considering the arguments presented above, it resulted that our case report was thoroughly grounded, based on objective data, analyzed, and corroborated with maximum diligence, in accordance with the highest national standards, as long as the criticisms made by Finsterer J. et al. are essentially unfounded. 

**Financial Disclosure**

None of the authors has any financial or proprietary interests to disclose.
